# Verification of Charge Transfer in Metal-Insulator-Oxide Semiconductor Diodes via Defect Engineering of Insulator

**DOI:** 10.1038/s41598-019-46752-1

**Published:** 2019-07-16

**Authors:** Donggun Lee, Jun-Woo Park, Nam-Kwang Cho, Jinwon Lee, Youn Sang Kim

**Affiliations:** 1 Program in Nano Science and Technology, Graduate School of Convergence Science and Technology, Seoul National University, 1 Gwanak-ro, Gwanak-gu, Seoul, 08826 Republic of Korea; 2 Samsung Display Company, Ltd, 181 Samsung-ro, Tangjeong-myeon, Asan-si, 31454 Chungcheongnam-Do Republic of Korea; 3 Advanced Institute of Convergence Technology, 145 Gwanggyo-ro, Yeongtong-gu, Suwon, 16229 Republic of Korea

**Keywords:** Electronic devices, Electrical and electronic engineering

## Abstract

In a MIS (Metal/Insulator/Semiconductor) structure consisting of two terminals, a systematic analysis of the electrical charge transport mechanism through an insulator is essential for advanced electronic application devices such as next-generation memories based on resistance differences. Herein, we have verified the charge transfer phenomenon in MIOS (Metal/Insulator/Oxide Semiconductor) diodes through a defect engineering of the insulator. By selectively generating the oxygen vacancies in the insulator (Al_2_O_3_), the MIOS diode rectification of the P^++^-Si anode/Al_2_O_3_/IGZO cathode reached 10^7^ at 1.8 V and considerably suppressed the leakage current. Studying the current-voltage characteristics of MIOS diodes shows that the charge carrier transport mechanism can vary depending on the defect density as well as the difference between the CBM (conduction band minimum) of the semiconductor and the oxygen vacancy energy level of the insulator.

## Introduction

Transient metal oxide materials have been highlighted due to their superior electrical and unique properties such as ease of thin film fabrication and high optical transparency. For these reasons, many researchers have made efforts into developing oxide electronic devices like TFTs (Thin Film Transistors) and TFDs (Thin Film Diodes), which are used as fundamental building blocks. Despite the significant research, while oxide TFTs were developed and commercialized, there still remain challenges to improving the oxide TFDs. As p-type oxide semiconductors show poor electrical properties, there are difficulties with making oxide p-n junction diodes^1,2^. Also, due to fermi level pinning between the metal-oxide semiconductor junction in the gap state, Schottky diodes are limited in electronic device applications^3,4^. In particular, most reported diodes have low rectifying ratio, which makes it difficult to control the leakage current. To solve these problems, we recently introduced MIOS (Metal-Insulator-Oxide Semiconductor) diodes^5^. Electronic devices that operate using the intrinsic trap in the insulator control the current magnitude and polarity by applying an oxide semiconductor electrode. Lee *et al*. have introduced the outstanding MIOS diode properties such as high rectifying ratio and low leakage current that overcome the disadvantages of conventional p-n junction diodes and Schottky diodes^5^. However, these early-stage MIOS diodes are highly dependent on the semiconductor area used for the cathode^6^. In order to apply MIOS diodes to the TFD in earnest, rigorous mechanism analysis of charge carrier transport through the insulator is required in addition to an accurate understanding of the insulator characteristics during device operation.

Herein, we successfully proved the charge transfer mechanisms of MIOS diodes by controlling the oxygen vacancy in the insulator, which is critical to its operation. This oxygen vacancy can be formed selectively within the Al_2_O_3_ (insulator) bandgap by working pressure variation defect engineering. During the Al_2_O_3_ layer deposition, the Ar background gas concentration was proportionally tailored to induce a controllability of the particle energy deposited on the substrate and form the vacancy defects according to atomic weight of target species. Consequently, the defect energy level and concentration in Al_2_O_3_ act as electrical paths that are formed selectively according to variations in the working pressure. This defect engineering of the insulator shows that the conduction mechanism of the MIOS diodes is closely related to the defect density as well as the energy level difference between the oxygen vacancy and the semiconductor CBM. Based on this correlation, we improved the MIOS diode performance such as high rectifying ratio and low voltage operation. Also, since there is no dependency on the cathode area, the MIOS diode can be applied to an actual TFD by showing the possibility of electric current flowing in a small top electrode. Subsequently, we demonstrated that the oxygen vacancies with (0/−1) and (−1/−2) charge transition levels affect the charge carrier transport of MIOS diodes by using XPS (X-ray Photoelectron Spectroscopy) and IETS (Inelastic Electron Tunneling Spectroscopy). Furthermore, we figured out how charge carriers move within Al_2_O_3_ based on the I-V curve extrapolation method and presented a current flow model in operation using the band-diagram. The capacity to transport a charge by selectively manipulating the energy level and density of oxygen vacancy will significantly enhance the performance of electrical devices with insulators such as next generation memory based on resistance differences.

## Results

### Electrical characteristics of MIOS diodes and defect engineering system

Defect engineering can optimize the physical and chemical properties of materials and is an indispensable element in the modern world of electronic devices. Recently, the insulating layers have been studied and developed as charge-transferring layers for transferring electrical charge carriers. Additionally, the defect concentration control in the insulating layer film has become extremely important. For example, ReRAM^7,8^ and vertically integrated NAND (V-NAND) flash memory^9^ devices control the defect concentration in the insulator film so that charge carriers can move through the insulator or be stored in the insulator. For this reason, many researchers have made efforts to develop various methods of defect engineering to optimize device performance. One of these is by doping other substances to the insulator, which forms additional states in the desired energy level within the energy band gap by adding appropriate impurity materials^10^. The second is by post treating the as-deposited insulator, which leads to extra chemical bonds between the elements in the insulator and the treatment gases including H_2_, O_2_, N_2_, and Ar^11,12^. The last is by controlling the reaction gas ratio during film growth, which causes variations in the stoichiometry of the metal and anion^13,14^. Although the above methods are efficient, they have some drawbacks like difficulty in adjusting the appropriate amount of the added material, high energy requirement during post treatment, and difficulty in precisely controlling the stoichiometry.

**Figure 1:**
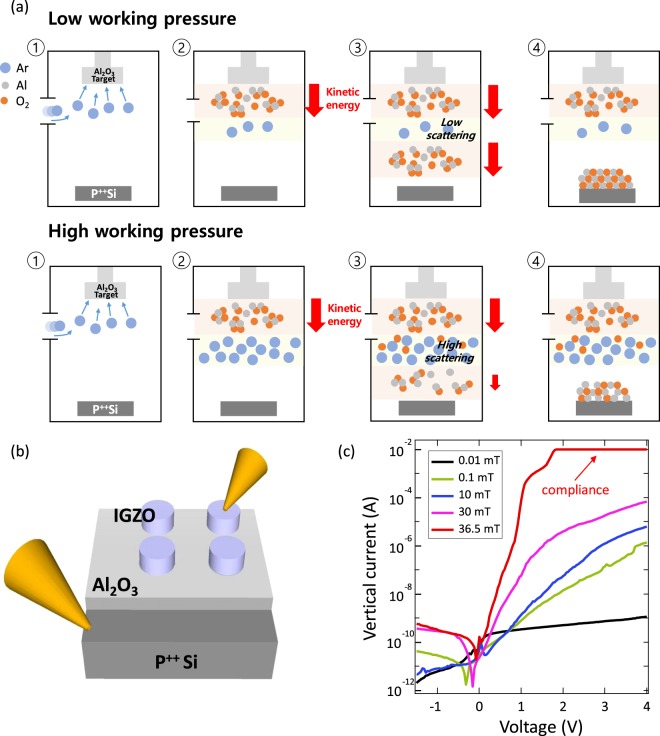
Schematic diagram of sputtering system and vertical transfer characteristic of Bottom electrode (anode)/Insulator/Oxide semiconductor (cathode) structure. (**a**) Schematic diagram of sputtering deposition system for working pressure variation defect engineering. (**b**) Schematic structure of the MIOS device consisting of P^++^-Si anode/insulator 10-nm Al_2_O_3_/20-nm IGZO cathode. (**b**) Vertical current-voltage characteristics of the MIOS diodes on the various condition; 0.01, 0.1, 10, 30, 36.5 mTorr.

One of the most common methods of thin film deposition is sputtering so that the film properties can be significantly influenced by the plasma process during the deposition stage. The physical properties of a film grown by sputtering are strongly dependent on the energy deposited by the particles reaching the substrate^15^. Teng *et al*. demonstrated the defect engineering via multi-step sputtering processes where they modulate the defect distribution and form controllable devices involving a Schottky diode and ReRAM for the first time^16^. Furthermore, M.Acosta *et al*. introduced the effect of weight on the target species where the number of collisions with Ar, a background gas, depends on the weight of the target species to form an oxygen vacancy^17^. As mentioned above, sputtering is a process that allows defect engineering to change material properties in a simple way. The above-mentioned characteristics can be implemented so that the working pressure of sputter can control the particle energy deposited on the substrate and form the vacancy defects according to atomic weight. Simple images that show the effect of working pressure variations are in Fig. 1(a). We proceeded with defect engineering by assuming that the working pressure variation effect is still valid regardless of materials.

Figure 1(b) shows the Metal/Insulator/Oxide Semiconductor (MIOS) device structure consisting of the P^++^-Si anode/Al_2_O_3_/IGZO cathode. The 10-nm-thick Al_2_O_3_ layer was deposited by a varying working pressure sputtering process on the highly boron-doped Si substrate. The 20-nm-thick IGZO cathode with a radius 250 µm was formed by a sputter (other experimental details are described in the method section). At first, we observed the I–V characteristics of the Metal/Insulator/Metal (MIM) structure to investigate the validity of the defect engineering according to the working pressure (0.01, 0.1, 10, 36.5 mTorr) during the sputtering process. Studying the MIM structure with the indirect electrical analysis method can examine the bonding strength and quality of the thin film by measuring the current flow in the insulator between the metals. We prepared the P^++^-Si/Al_2_O_3_ (10 nm)/Al (100 nm) MIM device in order to compare the electrical performance of Al_2_O_3_ layers made in various working pressures. Subsequently, a voltage was applied to the P^++^-Si anode using a two-probe system to measure the amount of the electrons passing through the Al_2_O_3_ thin film. Supplementary Fig. 1 is the schematic structure and I-V curve showing the leakage current as well as the breakdown strength of the MIM device depending on working pressure. According to the results, the working pressure rises and the breakdown strength decreases while the leakage current increases. Thus, the higher the working pressure, the more electrical traps or intrinsic defects act as leakage paths in Al_2_O_3_.

In order to make the MIOS diodes, we deposited the IGZO semiconductor with a radius 250 µm as the cathode instead of Al metal. The measured I-V characteristics of the MIOS diodes are shown in Fig. 1(c). Surprisingly, the MIOS diodes allow large amounts of electrical current to flow in a voltage range from −4 V to 4 V, only from the P^++^-Si anode to IGZO cathode, as the working pressure increases. As a result, the rectification ratio reached 10^7^ at 1.8 V, which is 10 times better than the previous case^5^, despite the considerably smaller cathode area. This means that MIOS diodes can be applied to the TFDs.

### Optical analysis and energy band diagram of Al_2_O_3_/IGZO heterogeneous films

**Figure 2:**
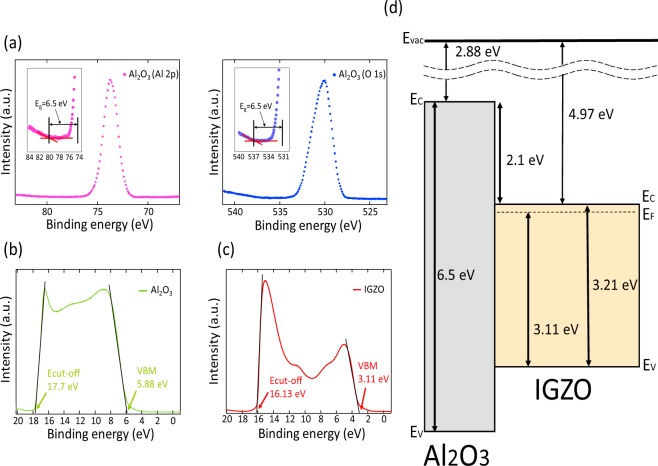
Optical analysis and energy band diagram of insulator-oxide semiconductor films. (**a**) XPS O 1s, Al 2p peak in Al_2_O_3_ made with 0.01 mTorr condition. The inset is the measurement of the band gap of 0.01 mTorr-Al_2_O_3_ using onset of electron energy loss spectra. (**b**) Entire UPS spectra of 0.01 mTorr-Al_2_O_3._ Cut-off and VBM energy of 10 mTorr-Al_2_O_3_ are 17.7 and 5.88 eV, respectively. (**c**) Entire UPS spectra of IGZO. Cut-off and VBM energy of IGZO are 16.13 and 3.11 eV, respectively. (**d**) Energy band diagram of Al_2_O_3_/IGZO heterogeneous films. CB offset between Al_2_O_3_ and IGZO is 2.1 eV. Electron affinity of Al_2_O_3_ and IGZO are 2.88 and 4.97 eV. Band gap of Al2O3 and IGZO are 6.5 and 3.21 eV.

To further understand the effect of defect engineering in MIOS diodes, we analyzed the electrical conduction based on the energy band diagram. In order to obtain the Al_2_O_3_ and IGZO band information including band gap, work-function, and VBM (Valence Band Maximum), we performed XPS (X-ray Photoelectron Spectroscopy), UV-vis spectroscopy, and UPS (Ultraviolet Photoelectron Spectroscopy). Figure 2(a) shows XPS spectra for the O 1 s and Al 2p peak in Al_2_O_3_ fabricated in the case of 0.01 mTorr. The band gap energy of Al_2_O_3_ was 6.5 eV, which is calculated by utilizing XPS via inelastic loss phenomenon^18^. These results are consistent with previous studies showing that amorphous Al_2_O_3_ has a band gap of 5.4 to 7.5 eV^19–21^. The band gap of Al_2_O_3_ is formulated under different working pressure conditions at 6.5 eV, indicating that the phase of the Al_2_O_3_ is also amorphous (Supplementary Fig. 2). Through the UV-vis Spectrum of IGZO (Supplementary Fig. 3), the optical band gap energy of IGZO (3.21 eV) is extracted by the Tauc plot. To obtain the work–function and VBM, we use the UPS. As a result, work-function of Al_2_O_3_ and IGZO were 3.51 and 5.07 eV while VBM were 5.95 and 3.11 eV, respectively. Figure 2(d) is an energy band diagram according to the results obtained from the above analysis (exact numerical calculations about optical analysis are in Supplementary Note 1).

### Electrical and chemical analysis of defective Al_2_O_3_ insulator

**Figure 3:**
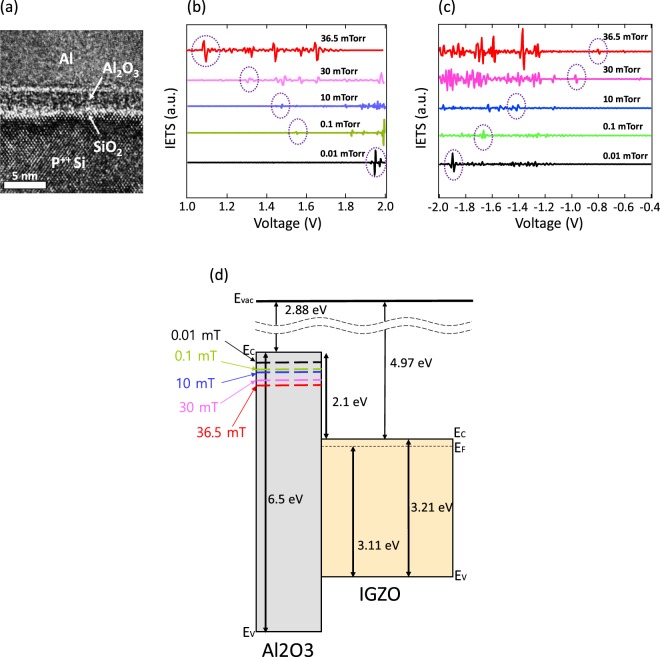
IETS spectrum and trap energy level within the Al_2_O_3_ band gap. (**a**) Cross-section TEM image of P^++^-Si/1.3-nm Al_2_O_3_ (made with 0.01 mTorr condition)/50-nm Al Metal-Insulator-Metal structure. (**b**,**c**) IETS spectra observed on Metal-Insulator-Metal structure according to various working pressure conditions. Circles represent the first trap related feature. (**b**) In the positive voltage region (**c**) In the negative voltage region (**d**) Trap energy level according to working pressure variation within Al_2_O_3_ band gap based on 0.01-mTorr-Al_2_O_3_.

To investigate the defect energy level within the Al_2_O_3_ band gap that acts as an electrical path way, we used the IETS (Inelastic Electron Tunneling Spectroscopy) method, which takes a second derivative of the current-voltage characteristics of tunnel barriers in the MIM or MIS structure. This is one of the precise analysis methods for carrier mobility, such as phonon mode and charge trap of a structure, which is difficult to accurately perceive when the insulator film is extremely thin. Since the charge trap signal has peak to valley or valley to peak characteristics that are distinguishable to phonon mode, we can accurately derive information about the trap. In addition, the defect energy level can be extracted by measuring the FTRF (First Trap Related Feature, V_f_ and V_r_; V_f_ is the applied voltage necessary to align the fermi level of the cathode with the trap energy level and V_r_ is the applied voltage necessary to align the fermi level of the anode with the trap energy level)^22^. For example, when the fermi level of the metal reaches the trap energy level of the insulator by the applied voltage, the current is represented by the sum of the direct tunneling current and the trap assisted conduction current. In order to analyze the IETS, we choose the MIM structure instead of MIS to avoid depletion of the semiconductor acting as electrical resistance. We deposited 1.3-nm-thick Al_2_O_3_ layers on the P^++^-Si substrate by sputtering at various working pressure conditions. To form the top electrode, 50-nm-thick Al was deposited by thermal evaporation for all samples. The size of the aluminum was 250 µm in diameter. Figure 3(a) show the cross-section TEM image of P^++^-Si/0.01 mTorr-Al_2_O_3_/Al MIM structure. It was confirmed that the thickness of Al_2_O_3_ is 1.3 nm and phase of Al_2_O_3_ is amorphous. In addition, we found a native oxide of about 0.7 nm, but we did not treat it as a variable parameter of the IETS analysis because we made the MIM device in the same substrate. Figure 3(b,c) are characteristics of the IETS spectrum in the forward and reverse bias. As shown in the figures, the FTRF values become zero as the working pressure increases, which means that the trap energy level of Al_2_O_3_ is different according to deposition conditions. Supplementary Table 2 shows V_t_, which indicates the trap energy level where the larger the working pressure, the lower the trap energy level in the Al_2_O_3_ band gap. Figure 3(d) shows the insertion of the trap energy into the band diagram obtained by optical analysis in order to intuitively understand the role of the trap. The higher the working pressure, the closer the trap energy level is to the conduction band of the semiconductor. At the same time, it is far away from the CBM of Al_2_O_3_. The higher the working pressure, the closer the trap energy level is to the semiconductor conduction band and at the same time away from the CBM of the Al_2_O_3_.

**Figure 4:**
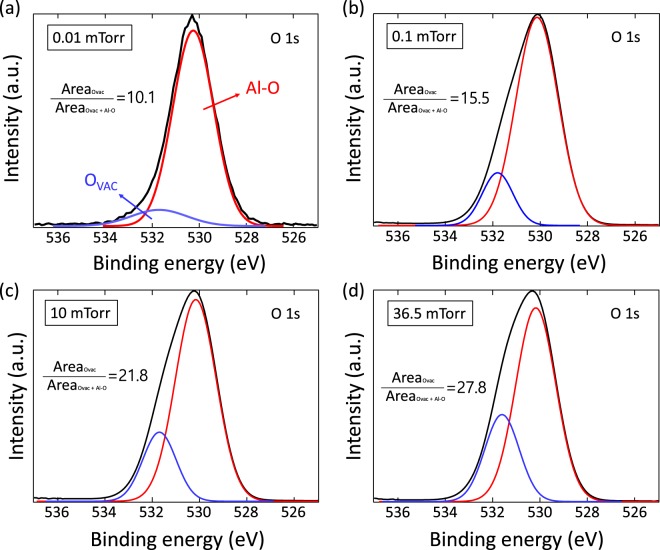
The O 1s spectra of Al_2_O_3_. XPS O 1s peak in Al_2_O_3_ made with (**a**) 0.01 mTorr (**b**) 0.1 mTorr (**c**) 10 mTorr (**d**) 36.5 mTorr.

To clarify what kind of defect is created in Al_2_O_3_, we analyzed the XPS spectrum to identify the chemical change of Al_2_O_3_. Figure 4 shows the O 1s spectrum results of Al_2_O_3_ at various working pressures. For further analysis, the O 1s spectrum is adjusted to the Gaussian-Lorentzian function. The O 1s spectrum can be divided into two main peaks: metal-oxygen bonding (O_Al-O_) with binding energy 530.17 eV and oxygen vacancy (O_vac_) with binding energy 531.8 eV^23^. The O_vac_/(O_Al-O_ + O_vac_) area ratios are 10.1 (0.01 mTorr), 15.5 (0.1 mTorr), 21.8 (10 mTorr), and 27.8 (36.5 mTorr) where the Al_2_O_3_ film fabricated at high working pressures has a higher oxygen vacancy. J. R. Weber *et al*. reported that the border traps or electrical leakage path at the III–V semiconductor/Al_2_O_3_ interface is oxygen vacancy through the hybrid density functional calculations of κ-Al_2_O_3_ phase, which are similar to the amorphous Al_2_O_3_^24^. The thermodynamic charge transition level for the oxygen vacancy of κ-Al_2_O_3_ to different coordinates of each defect is widely distributed from the semiconductor band gap to below the CBM of κ-Al_2_O_3_. D. Liu *et al*. also reported similar results in monoclinic θ-Al_2_O_3_ with sixfold and fourfold Al sites as well as both three and twofold oxygen sites^25^. Consequently, based on our experimental results and the theoretical calculations mentioned above, the working pressure variation defect engineering can selectively change the formation energy of oxygen vacancies by controlling the chemical potentials of Al and O_2_. Oxygen vacancies with (0/−1) and (−1/−2) can be generated at desired positions within the Al_2_O_3_ band gap.

### Charge-transport mechanisms in the MIOS diodes

**Figure 5:**
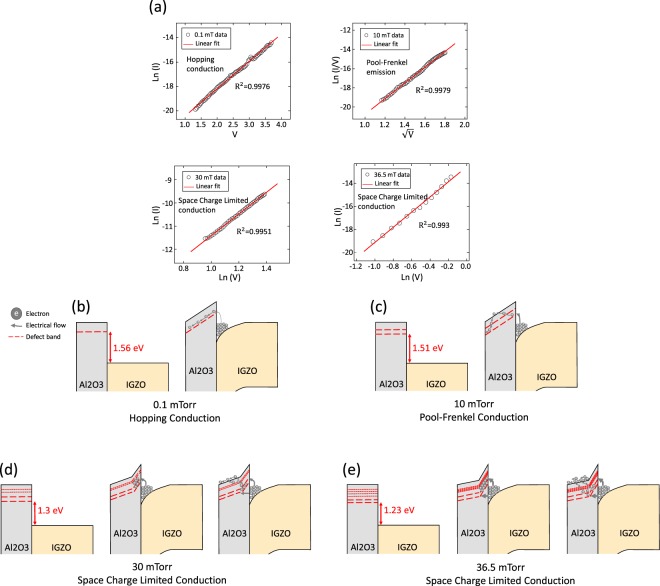
Current transport characteristics and schematic diagram of MIOS diodes operation based on electron flow. (**a**) The extrapolation results based on I–V characteristics through bulk-limited-conduction mechanism associated with defect when conduction occured;0.1 mTorr (1.3~3.6 V), 10 mTorr (1.3~3.2 V), 30 mTorr (0.5~2 V), 36.5 mTorr (0.4~0.9 V). (**b**–**e**) Modeling of defect induced current flow in MIOS diodes. (**b**) 0.1 mTorr: Hopping conduction (**c**) 10 mTorr: Pool-Frenkel conduction (**d**) 30 mTorr: SCLC (**e**) 36.5 mTorr: SCLC.

In order to obtain the energy band modeling of MIOS diodes according to trap energy level, we investigated the electrical conduction mechanism through the I–V curve analysis. Because there are a number of conduction mechanisms that can contribute to the conduction of charge carrier through the dielectric film at the same time, an accurate I–V fitting is important. Thus, we proceeded with the I-V fitting based on changes in the current amount (from 10^9^ to 10^6^ A) where conduction occurred, which are 0.1 mTorr (1.3~3.6 V), 10 mTorr (1.3~3.2 V), 30 mTorr (0.5~2 V), and 36.5 mTorr (0.4~0.9 V). Figure 5(a) represents the extrapolation results based on bulk-limited-conduction mechanism associated with defects in the insulator^26,27^. As a result, the I–V characteristics are fitted with Hopping, Pool-Frenkel, and SCLC Mechanism as the working pressure increases by 0.1, 10, 30, and 36.5 mTorr (the extrapolation results are in Supplementary Note 3). Based on the results, a schematic of the energy band diagram in the operating state is shown in Fig. 5(b). In the case of 0.1 mTorr, a relatively large amount of energy is required to move the electrons injected from the IGZO cathode to the electrical path in the insulator, because the V_t (0.1 mTorr)_ is closer to the CBM of Al_2_O_3_ than other conditions. For this reason, the MIOS diode mechanism in 0.1 mTorr is the hopping conduction, because there are only a few electrons injected from IGZO to Al_2_O_3_ despite the presence of a defect energy level. The V_t (10 mTorr)_ is closer to CBM of IGZO for 10 mTorr than for 0.1 mTorr. Consequently, the amount of electrons injected into Al_2_O_3_ increased and they transfer to the anode with Pool-Frenkel conduction. These results are similar to previous results which show that the conduction mechanism flowing through Al_2_O_3_ deposited by ALD when the CB (Conduction Band) Offset is 1.5 eV in the Al_2_O_3_/Si structure is Pool-Frenkel^28^. The conduction mechanism flowing through Al_2_O_3_ is assumed to not be dependent on how it is made, but only on a deep correlation to the trap energy level. However, there is a different tendency in the case of 30 mTorr in our analysis. As mentioned in the previous IETS results, it was confirmed that the Al_2_O_3_ trap energy states are widely distributed when the thin film is deposited at 30 mTorr or more. In addition, the electrons thermally excited at room temperature are injected into the Al_2_O_3_, because the V_t (30 mTorr)_ is closer to the CBM of IGZO. The compiled electrons momentarily create a large electric field on the Al_2_O_3_ near the cathode. Because of this effect, electrons under the larger electric field flow more easily through the CBM of Al_2_O_3_ and the conduction mechanism is SCLC. Furthermore, the SCLC mechanism dominates when it is 36.5 mTorr. The only difference between 30mTorr and 36.5mTorr is that the defect state acting as an electrical path is widely distributed so that the amount of current is amplified, and the electron is injected at a lower voltage because V_t (36.5mTorr)_ is located at the lowest energy level. This result shows that the defect density as well as the energy difference between the oxygen vacancy energy level in the insulator and the CBM of the semiconductor are closely related to the MIOS diode conduction mechanism.

## Conclusion

In summary, we validated the MIOS diodes electrical charge transfer mechanism through the defect engineering of insulator. By selectively forming the oxygen vacancies in the insulator, which are important for MIOS diodes operation, we clarified that the conduction mechanism of MIOS diodes is closely related to the oxygen vacancy density as well as the energy level difference between the oxygen vacancy and the CBM of semiconductor. Although the Al_2_O_3_ (insulator) has the same amorphous phase, it is possible to control the conduction band off-set between insulator and contact material by forming the oxygen vacancy state at a desired location within the band gap through defect engineering. Due to these effects, we successfully improved the performance of MIOS diodes such as high rectifying ratio and low voltage operation to result in 10^7^ at 1.8 V. In addition, we found the oxygen vacancies with (0/−1) and (−1/−2) charge transition levels play important roles in the charge carrier transport of MIOS diodes by using XPS and IETS. Furthermore, we proposed how charge carriers move within Al_2_O_3_ based on the energy band diagram according to the energy level and density of the oxygen vacancy as well as conduction mechanism of MIOS diodes could be understood in depth. We believe that this strategy not only helps to improve the electrical performance of oxide electronic devices, but also solve the electronic device problem by implementing various functions using defects.

## Method

### Devices preparation

A 525-µm-thick, highly boron-doped, single-side-polished, (100)-oriented, P^++^-type Si wafer with a resistivity of <0.005 Ω·cm was first cleaned sequentially with detergent in an ultrasonic bath for 15 min, and de-ionized (DI) water for 20 min. To remove organic residues, P^++^-Si wafer was cleaned with acetone and isopropyl alcohol (IPA) in an ultrasonic bath for 15 min, respectively. Finally, the prepared wafer was dried by N_2_ blowing. And the size of all specimens was 2 × 2 cm^2^.

Thin 10-nm-Al_2_O_3_ insulator layer was deposited on P^++^-Si anode layer by using RF magnetron sputtering system (DAEKI HI-TECH Co.,Ltd, Co-Sputtering System) with a planer round insulator 3-inch-Al_2_O_3_ target (purity >99.99%) under 10^−4^ mTorr at room temperature by power of 150 W. Only the pure inert gas, Ar (purity >99.99%), was used to fix the chamber pressure as a variable, and the pressure of chamber was adjusted from 10^−4^ to 0.01, 0.1, 10,30 and 36.5 mTorr during sputtering. Subsequently, 20-nm-IGZO cathode layer was deposited on Al_2_O_3_ insulator layer by RF magnetron sputtering 3inch-IGZO target (In:Ga:Zn:O = 1:1:1:4 at%) in mixed Ar/O_2_ atmosphere at room temperature by power of 70 W. The area of the semiconductor was patterned using circle-shaped SUS shadow masks with diameter 250 µm. The thickness of Al_2_O_3_ and IGZO deposited by the sputtering system were measured using as α-step (KLA Tencor Co., Ltd, Alpha-step IQ surface profiler) and ellipsometer system (K-MAC Co.,Ltd, ST-2000 DLXn).

To fabricate the MIM (Metal-Insulator-Metal) devices, 100-nm-Al metal was deposited instead of a semiconductor by using thermal evaporator system (DAEKI HI-TECH CO.,Ltd, Thermal Evaporation System) under 10^−4^ mTorr on the Al_2_O_3_ insulator layer made in various working pressure condition; 0.01, 0.1, 10, 30 and 36.5 mTorr. The area of Al top electrode was patterned using circle-shaped SUS shadow masks with diameter 250 µm.

### Characterization of the fabricated devices

The current-voltage characteristics for all devices were measured using an Agilent 4155B semiconductor parameter analyzer with compliance current 10^−2^ A at room temperature in the dark.

## Supplementary information

Supporting information
