# Supplementary material for: Verification of Charge Transfer in Metal-Insulator-Oxide Semiconductor Diodes via Defect Engineering of Insulator
Source: Sci Rep. 2019 Jul 16;9:10323. doi: 10.1038/s41598-019-46752-1 (PMC6635483; doi:10.1038/s41598-019-46752-1)
Supplement: Supplementary file 1 — Supporting information [file 41598_2019_46752_MOESM1_ESM.docx]

Supplementary information

**Verification of charge transfer in metal-insulator-oxide semiconductor diodes via defect engineering of insulator**

*Donggun Lee*^1^**^†^**, *Jun-Woo Park*^1^**^†^**, Nam-Kwang Cho^1^, *Jinwon Lee*^2^* *& Youn Sang Kim*^1,3^*

^1^Program in Nano Science and Technology, Graduate School of Convergence Science and

Technology, Seoul National University, 1 Gwanak-ro, Gwanak-gu, Seoul 08826, Republic of

Korea

^2^Samsung Display Company, Ltd, 181 Samsung-ro, Tangjeong-myeon, Asan-si, Chungcheongnam-Do, Republic of Korea

^3^Advanced Institute of Convergence Technology, 145 Gwanggyo-ro, Yeongtong-gu, Suwon

16229, Republic of Korea

*Corresponding author; Youn Sang Kim (E-mail: younskim@snu.ac.kr, tel: 82-31-888-9131)

Jinwon Lee (E-mail: [john.lee@samsung.com](mailto:john.lee@samsung.com))

†Co-author: Donggun Lee & Jun-Woo Park

These authors contributed equally to this work.

**Supplementary Figures**

Supplementary Figure 1: Schematic diagram of MIM device and electrical characteristic of Bottom electrode (anode) /Insulator/Oxide semiconductor (cathode) structure. (a) Schematic structure of the MIM device consisting of P^++^-Si anode/insulator 10-nm Al_2_O_3_/100-nm Al cathode. (b) Current density-Electric field characteristics of the MIM diodes on the various condition; 0.01, 0.1, 10, 30, 36.5 mTorr and ALD.

Supplementary Figure 2: XPS O 1s, Al 2p peak in Al­_2_O_3_ (a) 10 mTorr (b) 30 mTorr (c) 36.5- mTorr. The inset is the measurement of the band gap using onset of electron energy loss spectra.

**Supplementary Notes**

**Supplementary Note 1. The exact numerical calculation of optical analysis in Al_2_O_3_/IGZO heterogeneous films.**

In order to obtain the band structure, Φ (work-function), E_V_ (valence band maximum), and E­_g_ (energy band gap) are essential. To acquire exact data, we investigated through optical analysis such as UPS (Ultraviolet Photoelectron Spectroscopy), XPS (X-ray Photoelectron Spectroscopy), and UV-vis spectrum. Fig S.4 shows the transmittance of IGZO range from 300-850 nm by using UV-vis spectrum so that the E_g_ (3.21 eV) is extracted by the tauc`s plot using equation (1).^1^

(αhν)^m^=A(hν-E_g_) ------ (1)

(α: Absorption coefficient of material, h: Flank constant, ν: frequency of light, m: nature of the transition) We select value of m is 2 because IGZO known as a direct band gap material.^1^

Supplementary Figure 3: UV-vis spectrum analysis of IGZO (a) The optical transmittance of IGZO in the range of 300 to 850 nm. (b) the (αhν)^2^ versus the photon energy plot of IGZO.

It is difficult to use the UV-vis spectrum in the case of Al_2_O_3_ since the E_g_ value of Al_2_O_3_ is 6~8 eV. Thus, we measured Al_2_O_3_ using XPS, which can cause inelastic loss phenomena.^2^ Based on this method, the E_g_ (6.5 eV) value of Al_2_O_3_ made at 0.01 mTorr was extracted. (Fig.2 (a)) and we used UPS to obtain Φ and E_V_. Before we measured, semiconductors and insulators were calibrated using Au metal plates due to low conductivity. Fig. 4 (b), (c) show the UPS data of Al­_2_O_3_ and IGZO after calibration. Usually, E_V_ can be measured by extrapolating the electron distribution curves decaying toward the band edge while the work-function can be obtained by calculating (E_incident photon_ - E_cut-off_).^3^

**Supplementary Note 2. IETS analysis of Al_2_O_3_ fabricated under working pressure variation condition.**

Fig. S3 shows the schematic diagram of the P^++^-Si/Al_2_O_3_/Al MIM structure to analyze IETS. Fig. S3 (a) shows a very thin Al_2_O_3_ sandwiched between Al and P^++^-Si electrodes with zero bias voltage and the fermi energy level of the two metals is the same. Fig. S3 (b) shows that the fermi energy level of Al and the defect energy level of Al_2_O_3_ are the same. Moreover, electrons injected from the Al electrode are trapped at the defect site and then moved to the opposite electrode. This series of processes makes the peak-to-valley or valley-to-peak as the charge trap signal and we can extract the applied voltage necessary to align the fermi level of the Al with the energy level of the defect, V­_f_. In this manner, we can measure the applied voltage necessary to align the fermi level of the P^++^-Si with the energy level of the defect, V_r_. Fig. S3 (c) shows that the fermi energy level of P^++^-Si coincides with the defect energy level of Al_­2_O_3_ where the injected electrons are trapped at the defect site and then transferred to the Al electrode. V_t_, the defect energy level, can be obtained using Equation (2) derived by T.P. Ma et al.^4^

V_t_ = - V_f_ x V_r_ / (V_f_ – V­_r_) ------ (2)

Table 2 shows the results of V_t ­­­_according to the working pressure variation for the MIM structure. In order to analyze through IETS, we applied the MIM structure instead of MIS to avoid semiconductor depletion acting as electrical resistance. We deposited 1.3-nm-thick Al_2_O_3_ layers on the P^++^-Si substrate by sputtering at various working pressure conditions (0.01, 0.1, 10, 30, and 36.5 mTorr). To form the top electrode, 50-nm-thick Al was deposited by thermal evaporation for all samples and the aluminum was 250 µm in diameter.

Supplementary Figure 3: Simplified schematic diagram of IETS analysis of P^++^-Si/1.3-nm-Al_2_O_3_/Al MIM Structure (a) Zero bias (b) Forward bias (c) Reverse bias

**Supplementary Note 3. Charge-transport mechanisms in the MIOS diodes**

There are two main types of conduction mechanisms through which current flows through an insulator: an electrode limited conduction mechanism and a bulk limited conduction mechanism. The electrode limited conduction mechanism depends on the interface properties between the insulator and the electrode junction such as the insulator thickness as well as the energy barrier height between the insulator and the electrode at the interface. In addition, the bulk limited conduction mechanism depends on the intrinsic properties of the insulator rather than the interface such as energy level and trap density. The bulk limited conduction mechanism mainly consists of three transfer mechanisms: (1) hopping conduction, (2) Pool-Frenkel conduction, and (3) Space-Charge-Limited Conduction (SCLC).^5^

The hopping conduction is caused by a tunneling effect by hopping the trap sites inside the insulator and can be expressed as Equation (3). If the slope is linear in the log (j) versus E graph, current flows through the hopping conduction according to Eq. (3).

$J=qanv exp[\frac{qaE}{kT}-\frac{E_{a}}{kT}$] ------ (3)

(q: electronic charge, a: mean hopping distance, n: electron concentration, v: frequency of thermal vibration of electrons at trap site, E: electric field across the dielectric, E_a_: activation energy, k: Boltzmann`s constant, T: absolute temperature)

Pool-Frenkel conduction is a transfer mechanism similar to the Shockey Mission Where the charge carriers in the trap site inside the insulator are excited by thermal energy and emit to the conduction band of the insulator. The Pool-Frenkel conduction can be expressed by Equation (4) where if the slope is linear in the ln (J / E) versus √E graph, current flows into the Pool-Frenkel conduction.

$J=q\mu N_{c}E exp[\frac{-q(\phi_{T}-\sqrt{\frac{qE}{\pi\varepsilon_{i}\varepsilon_{0}}})}{kT}]$ ------ (4)

(µ: electronic drift mobility, N_C_: density of states in the conduction band, qΦ_T_ :trap energy level, ε_ox_: relative permittivity of dielectric oxide, ε_0_: permittivity of the free space)

The SCLC conduction is a three-step process. In the first step, the electrons are injected ohmically into the trap site of the insulator. Then, the injected electrons fill the emptied trap sites and accumulate inside the insulator before finally a space charge builds up and a strong electric field is formed where the injected charge carriers move freely in the insulator. Equation (5) can be represented as the child`s raw equation related to space charge limited conduction.^6^ According to Eq. (5), if the slope is linear in the ln (J) versus ln E graph, the current flows into space charge limited conduction.

$J=\frac{9}{8}\mu\varepsilon_{0}\varepsilon_{ox}\frac{V^{2}}{t_{ox}^{3}}$ ------ (5)

(V: applied voltage, t_ox_: thickness of dielectric)

To obtain the conduction mechanism according to working pressure variation defect engineering, we investigate the electrical conduction mechanism analysis based on bulk limited conduction mechanism by using equations (3), (4), and (5). Fig. 5(a) shows the R^2^ value obtained by extrapolating equations (3), (4), and (5) to the I-V curve. The R^2^ value indicates the extent to which the expected value of the trend line matches the actual data with a value between 0 and 1. Consequently, the closer to 1, the more stable.

**Supplementary Table**

**Supplementary Table 1.** The breakdown strength at 10^-4^ A and leakage current at 2 MV/cm of each 0.01~36.5 mTorr and ALD Al_2_O_3_ films.

|  | ALD | 0.01 mT | 0.1 mT | 10 mT | 30 mT | 36.5 mT |
| --- | --- | --- | --- | --- | --- | --- |
| Breakdown strength (MV/cm) | 8.24 | 6.28 | 3.4 | 1.4 | 0.6 | 0.32 |
| Leakage current (A/cm^2^) | 3.95 x 10^-8^ | 1.31 x 10^-7^ | 2.13 x 10^-6^ | 7 x 10^-4^ | 1.22 | 4.99 |

**Supplementary Table 2.** The first trap related feature results of 0.01~36.5 mTorr Al_2_O_3 ­_films obtained using P^++^-Si/1.3-nm-Al_2_O_3_/Al MIM structure.

|  | 0.01 mT | 0.1 mT | 10 mT | 30 mT | 36.5 mT |
| --- | --- | --- | --- | --- | --- |
| V_f_ (V) | 1.95 | 1.5 | 1.45 | 1.3 | 1.1 |
| V_r_ (V) | -1.9 | -1.65 | -1.5 | -0.9 | -0.8 |
| V_t_ (V) | 0.96 | 0.79 | 0.74 | 0.53 | 0.46 |

**Supplementary Table 3.** The ratio of Ovac bond area to (Ovac + M-O) bond area and Ovac bond area to M-O bond area by varying working pressure.

|  | 0.1 mT | 10 mT | 36.5 mT |
| --- | --- | --- | --- |
| Area_Ovac_ / Area_Al-O + Ovac_ | 15.5 | 21.8 | 27.8 |
| Area_Ovac_ / Area_Al-O_ | 18.3 | 27.8 | 38.5 |

**Supplementary Reference**

1 Hsu, C.-M., Tzou, W.-C., Yang, C.-F. & Liou, Y.-J. Investigation of the high mobility IGZO thin films by using co-sputtering method. *Materials* **8**, 2769-2781 (2015).

2 Nichols, M. *et al.* Measurement of bandgap energies in low-k organosilicates. *Journal of Applied Physics* **115**, 094105 (2014).

3 Klein, A. *et al.* Transparent conducting oxides for photovoltaics: Manipulation of fermi level, work function and energy band alignment. *Materials* **3**, 4892-4914 (2010).

4 Wang, M., He, W. & Ma, T. Electron tunneling spectroscopy study of traps in high-k gate dielectrics: Determination of physical locations and energy levels of traps. *Applied Physics Letters* **86**, 192113 (2005).

5 Chiu, F.-C. A review on conduction mechanisms in dielectric films. *Advances in Materials Science and Engineering* **2014** (2014).

6 Lee, J., Lim, K.-H. & Kim, Y. S. Effects of Unusual Gate Current on the Electrical Properties of Oxide Thin-Film Transistors. *Scientific reports* **8**, 13905 (2018).
